# Comparative Evaluation of Prognostic Scoring Systems for Metastatic Spinal Tumors: Utility of the New Katagiri and Skeletal Oncology Research Group Models

**DOI:** 10.3390/jcm15207780

**Published:** 2026-10-09

**Authors:** Junya Miyahara, Kazuki Abe, Rie Kodama, Masanori Fujiwara, Takuma Koyama, Takahiro Hozumi, Toshihide Hirai, Shurei Sugita

**Affiliations:** 1 Department of Orthopaedic Surgery, Tokyo Metropolitan Komagome Hospital, Bunkyo 113-8677, Tokyo, Japan; 2 Department of Orthopaedic Surgery, The University of Tokyo Hospital, Bunkyo 113-8655, Tokyo, Japan; 3 Department of Musculoskeletal Oncology, Tokyo Metropolitan Komagome Hospital, Bunkyo 113-8677, Tokyo, Japan

**Keywords:** metastatic spinal tumor, prognostic scoring system, New Katagiri score, SORG classic scoring algorithm, SORG machine learning algorithm

## Abstract

**Background**: Prognostic models for metastatic spinal tumors have evolved from conventional point-based scores to models incorporating treatment, laboratory, and machine-learning variables. However, comparisons in contemporary Japanese surgical cohorts remain limited. **Methods**: This retrospective study included 171 of 208 consecutive patients who underwent surgery for metastatic spinal tumors between January 2017 and August 2024 and had ascertainable 1-year survival status. Seven prognostic systems were compared using receiver operating characteristic area under the curve (AUC) analysis for 90-day and 1-year survival. Pairwise AUC comparisons were adjusted using the Holm method. Calibration and Brier scores were also assessed for the Skeletal Oncology Research Group (SORG) machine learning algorithm (MLA). **Results**: For 90-day survival, AUCs were 0.77 (95% confidence interval [CI], 0.58–0.95) for the SORG MLA and 0.76 (95% CI, 0.60–0.91) for the New Katagiri score. For 1-year survival, AUCs were 0.79 (95% CI, 0.72–0.86) for the New Katagiri score, 0.76 (95% CI, 0.68–0.83) for the SORG classic scoring algorithm, and 0.74 (95% CI, 0.66–0.82) for the SORG MLA. After Holm adjustment, no significant pairwise differences were found among these three models at either prediction horizon. SORG MLA calibration deviated from perfect calibration. **Conclusions**: In this single-center surgical cohort, the New Katagiri score and SORG models showed higher discrimination than several conventional scoring systems. However, these findings do not establish model equivalence or the clinical benefit of model-guided decisions. Broader external validation and assessment of clinical utility are needed.

## 1. Introduction

Bone is the third most common site of metastasis after the lungs and liver, with bone metastases particularly common in patients with advanced breast and prostate cancers [1,2]. The spine is an important site of skeletal metastasis because spinal involvement can cause substantial morbidity, including vertebral collapse and metastatic spinal cord compression [3]. Approximately 70% of bone metastases involve the spine [4], and about 10% of patients with spinal metastases develop pain or neurological deficits requiring consideration of surgery with radiotherapy [4,5]. Given the invasiveness of surgery, reliable estimation of life expectancy is crucial for selecting appropriate candidates [6,7,8].

Several prognostic scoring systems have been developed to estimate survival and guide treatment selection. Earlier systems, including the modified Bauer [9], revised Tokuhashi [10], and Tomita scores [11], primarily consider primary tumor type, performance status, metastatic burden, neurological status, and visceral metastases [12]. Although these systems have supported clinical decision-making, most were developed before the widespread use of molecularly targeted therapies, immune checkpoint inhibitors, and contemporary high-precision radiotherapy. Advances in systemic and local therapies have prolonged survival in patients with metastatic spinal tumors, raising concerns that traditional prognostic systems may underestimate survival in contemporary populations [13,14,15,16,17].

To address these limitations, more recent prognostic models have incorporated variables that better reflect contemporary oncological practice. The New Katagiri score incorporates primary tumor characteristics, visceral or cerebral metastases, laboratory abnormalities, performance status, previous chemotherapy, and skeletal metastatic burden; its classification of primary tumors also accounts for the availability of molecularly targeted therapy for lung cancer [13]. The Skeletal Oncology Research Group (SORG) models incorporate a broader range of clinical and laboratory variables [18]. The SORG machine learning algorithm (MLA) uses machine-learning techniques to generate individualized probabilities of 90-day and 1-year survival rather than assigning patients to broad prognostic categories [19]. External validation studies have shown generally favorable discrimination of the SORG models in contemporary and international cohorts [20,21], although calibration differences across populations have been reported [22].

Despite the increasing number of prognostic models, external validation and head-to-head comparisons of traditional and contemporary systems in Japanese patients remain limited [15,23]. Moreover, discrimination alone does not establish calibration or clinical usefulness, and model performance may vary across healthcare systems and tumor populations. Therefore, this study compared the discriminative performance of seven representative prognostic systems in a contemporary Japanese surgical cohort and additionally assessed calibration and overall prediction error for the probability-based SORG MLA.

## 2. Materials and Methods

This retrospective observational study screened consecutive patients who underwent surgery for metastatic spinal tumors at our hospital between January 2017 and August 2024. Surgical indications were generally based on one or more of the following: severe movement-related pain or neurological deficits attributable to spinal metastases, epidural spinal cord compression of Bilsky grade ≥ 2 [24], or spinal instability defined as a Spinal Instability Neoplastic Score (SINS) ≥ 7 [25]. The final decision regarding the indication for surgery and the surgical procedure was made by consensus within our department. Of the 208 patients who underwent surgery, 37 were alive but had <1 year of follow-up, mainly because they had been transferred to other hospitals or institutions. These patients were excluded because their 1-year survival status could not be determined for the binary receiver operating characteristic (ROC) analysis. Patients who died within 1 year were retained because their 1-year survival status was known. The remaining 171 patients comprised the analytical cohort (Supplementary Figure S1). This outcome-dependent eligibility criterion ensured ascertainment of the binary 1-year endpoint but may have introduced selection bias, as discussed below. Because all eligible patients during the predefined study period were included, no a priori sample size calculation was performed. Clinical variables included age, sex, body mass index (BMI), American Society of Anesthesiologists Physical Status (ASA-PS), Eastern Cooperative Oncology Group (ECOG) performance status, Karnofsky Performance Status, Frankel grade, previous systemic therapy, primary tumor type, numbers of spinal and extraspinal bone metastases, and visceral or brain metastases. Routine preoperative laboratory variables included white blood cell, neutrophil, and lymphocyte counts; hemoglobin; platelet count; creatinine; bilirubin; alkaline phosphatase; lactate dehydrogenase; calcium; potassium; C-reactive protein; and prothrombin time-international normalized ratio.

Seven prognostic scoring systems were evaluated: (1) SORG MLA [19], (2) SORG classic scoring algorithm [18], (3) New England Spinal Metastasis Score (NESMS) [26], (4) modified Bauer score [9], (5) New Katagiri score [13], (6) revised Tokuhashi score [10], and (7) Tomita score [11]. Variables, coding rules and direction of interpretation for the six point-based systems are summarized in Supplementary Tables S1–S6. Predicted probabilities from the SORG MLA were obtained using the web-based calculator cited in the original publication [19,27], without refitting or recalibration to the present cohort. Predictors were defined using the most recent information available before surgery. Because of the retrospective design, investigators calculating the scores were not formally blinded to postoperative survival. All variables required for the seven models were available for all 171 patients. Therefore, no imputation was required, and all models were evaluated in the same cohort.

The primary outcomes were discrimination for postoperative 90-day and 1-year survival. Survival status at these time points was retrospectively ascertained from electronic medical records. Survival time was calculated from surgery to death or the last follow-up. For binary ROC analyses, survival was the event of interest. Vital status at both prediction horizons was ascertainable for all 171 patients; therefore, no patient was censored before either ROC time point. Overall survival and survival probabilities at 90 days and 1 year were estimated using the Kaplan–Meier method with 95% confidence intervals (CIs). Median follow-up duration was estimated using the reverse Kaplan–Meier method with 95% CIs. ROC curves and areas under the curve (AUCs) with 95% CIs were calculated for each model. Pairwise AUC comparisons among all seven systems were performed using DeLong’s test, resulting in 21 comparisons at each prediction horizon. To control family-wise error from multiple comparisons, *p* values were adjusted using the Holm method separately for the 90-day and 1-year analyses. A Holm-adjusted *p* < 0.05 was considered statistically significant. AUCs were interpreted using point estimates and 95% CIs rather than fixed qualitative categories.

For the probability-based SORG MLA, calibration and overall predictive accuracy were assessed for 90-day and 1-year survival using calibration plots, calibration intercepts and slopes, and Brier scores. The 95% CIs for the Brier scores were estimated using the percentile method with 2000 nonparametric bootstrap resamples. Equivalent calibration analyses were not performed for the point-based systems because these systems do not uniformly provide individual predicted probabilities at these time points without model-specific transformation or refitting.

Descriptive statistics were used to summarize the analytical cohort. For the prespecified comparison of baseline characteristics between included and excluded patients, Fisher’s exact test was used for categorical variables and Student’s *t*-test for continuous variables after assessing distributional assumptions. A two-sided *p* < 0.05 was considered statistically significant for this comparison. All analyses except DeLong’s test and Holm adjustment were performed using JMP version 18.0.0 (SAS Institute, Inc., Cary, NC, USA); DeLong’s test and Holm adjustment were performed using R version 4.6.1.

## 3. Results

### 3.1. Patient Characteristics and Survival Outcomes

The final analysis included 171 patients with a mean age of 64.0 ± 13.5 years; 97 (56.7%) were male. ASA-PS ≥ 3 was present in 3.5%, ECOG performance status ≥ 3 in 32.7%, and Frankel grade C or D in 59.1%. Previous systemic therapy had been administered in 24.6%, and visceral or brain metastases were present in 35.9% (Table 1). Of the 171 patients, 6 underwent decompression alone and 165 underwent spinal fixation with or without decompression, including 7 who underwent total en bloc spondylectomy. Seven patients underwent emergency surgery within 24 h after the decision to operate. Postoperative systemic therapy was administered to 90 patients, and postoperative radiotherapy was administered to 135 patients. Among the 37 patients excluded because their 1-year survival status could not be ascertained, the median postoperative follow-up was 2 months (95% CI, 1–4 months), and all were alive at the last contact. Their baseline characteristics are summarized in Table A1. The excluded group had a higher proportion of patients with ECOG performance status ≥3 than the included group (70.2% vs. 32.7%, *p* < 0.001), indicating that exclusion may have altered the case mix.

Mean preoperative laboratory values were generally within normal clinical ranges. However, C-reactive protein levels were mildly elevated (1.4 ± 2.9 mg/dL), reflecting systemic inflammation in a subset of patients (Table 2).

Kidney cancer was the most common primary malignancy (16.4%), followed by lung cancer (14.0%), thyroid cancer (9.9%), sarcoma (9.9%), gastrointestinal cancer (8.8%), breast cancer (8.2%), and prostate cancer (7.0%) (Table 3).

**Figure 1:**
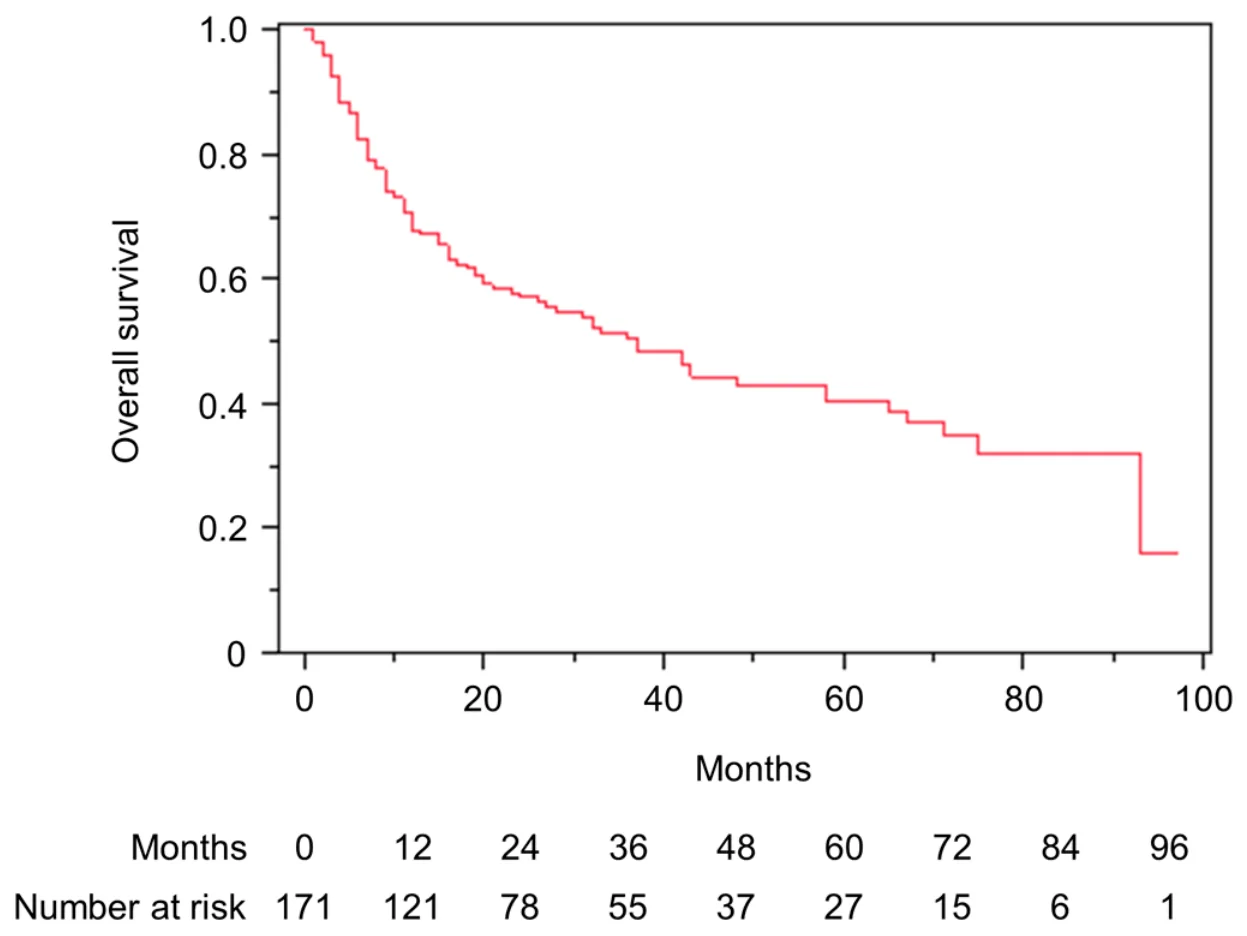
Kaplan–Meier curve for patient survival after surgery.

At 90 days after surgery, 7 patients (4.1%) had died and 164 (95.9%) were alive; at 1 year, 50 patients (29.2%) had died and 121 (70.8%) were alive. At the time of analysis, 94 of 171 patients (55.0%) had died and 77 (45.0%) were censored. The median follow-up duration, estimated using the reverse Kaplan–Meier method, was 45 months (95% CI, 39–59 months). Kaplan–Meier analysis demonstrated a median overall survival of 37 months (95% CI, 23–58 months). The estimated survival probabilities were 95.9% (95% CI, 91.6–98.0%) at 90 days and 70.8% (95% CI, 63.3–77.0%) at 1 year (Figure 1).

### 3.2. Comparison of Prognostic Scoring Systems

**Figure 2:**
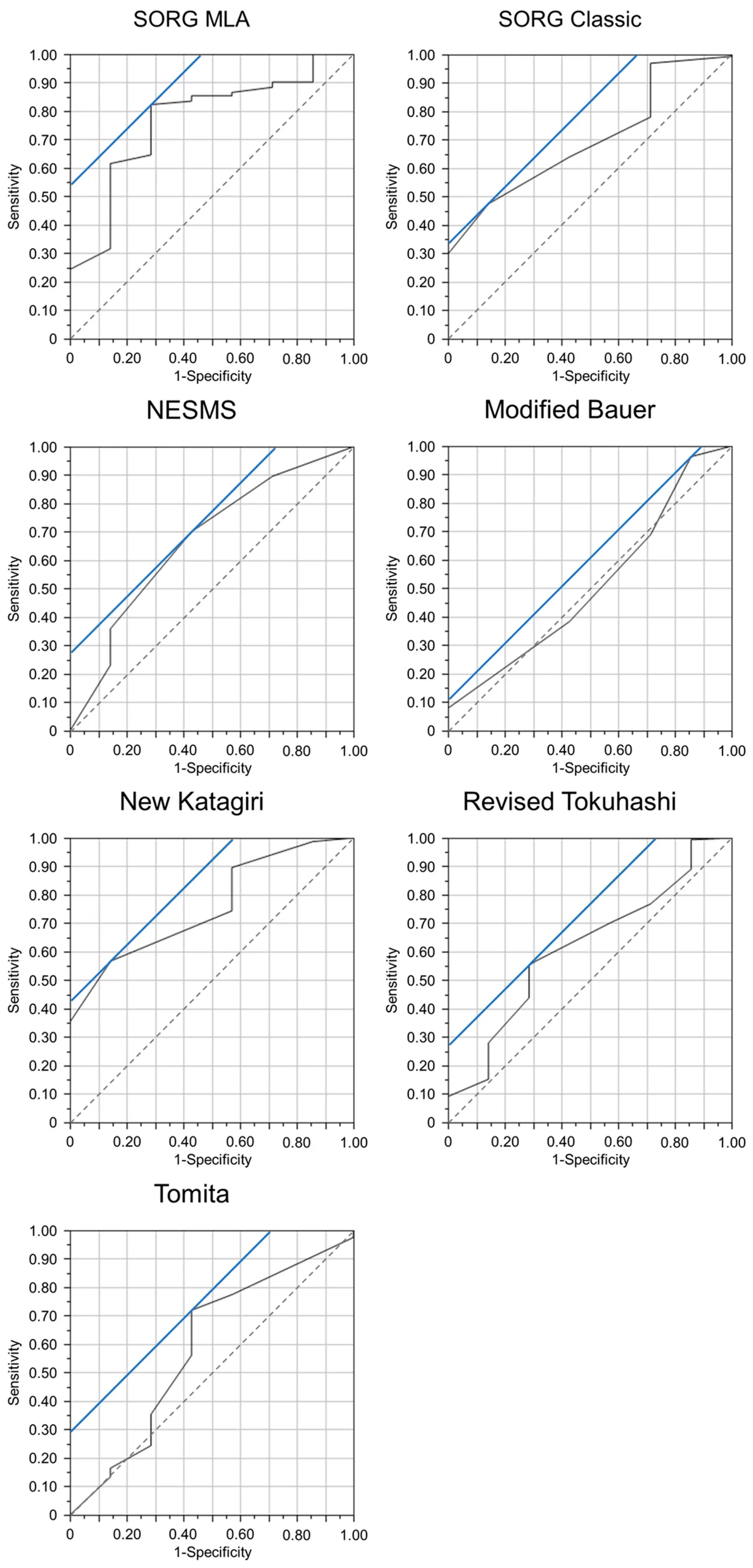
ROC curves for seven prognostic scoring systems for 90-day survival. Higher predictor values indicate a greater probability of survival. The black line represents the ROC curve, and the blue line is tangent to the ROC curve at the optimal cutoff point. The dashed diagonal line represents chance-level discrimination (AUC = 0.50).

Prognostic scores were calculated for each patient for ROC analysis, and their distributions are shown in Supplementary Figure S2. For 90-day survival, the SORG MLA had the highest AUC (0.77; 95% CI, 0.58–0.95), followed by the New Katagiri score (0.76; 95% CI, 0.60–0.91) and the SORG classic scoring algorithm (0.70; 95% CI, 0.53–0.87) (Table 4, Figure 2). AUCs for the remaining models ranged from 0.51 to 0.67. None of the 21 pairwise DeLong comparisons was statistically significant after Holm adjustment (Table A2). The wide CIs indicate limited precision at this prediction horizon.

**Figure 3:**
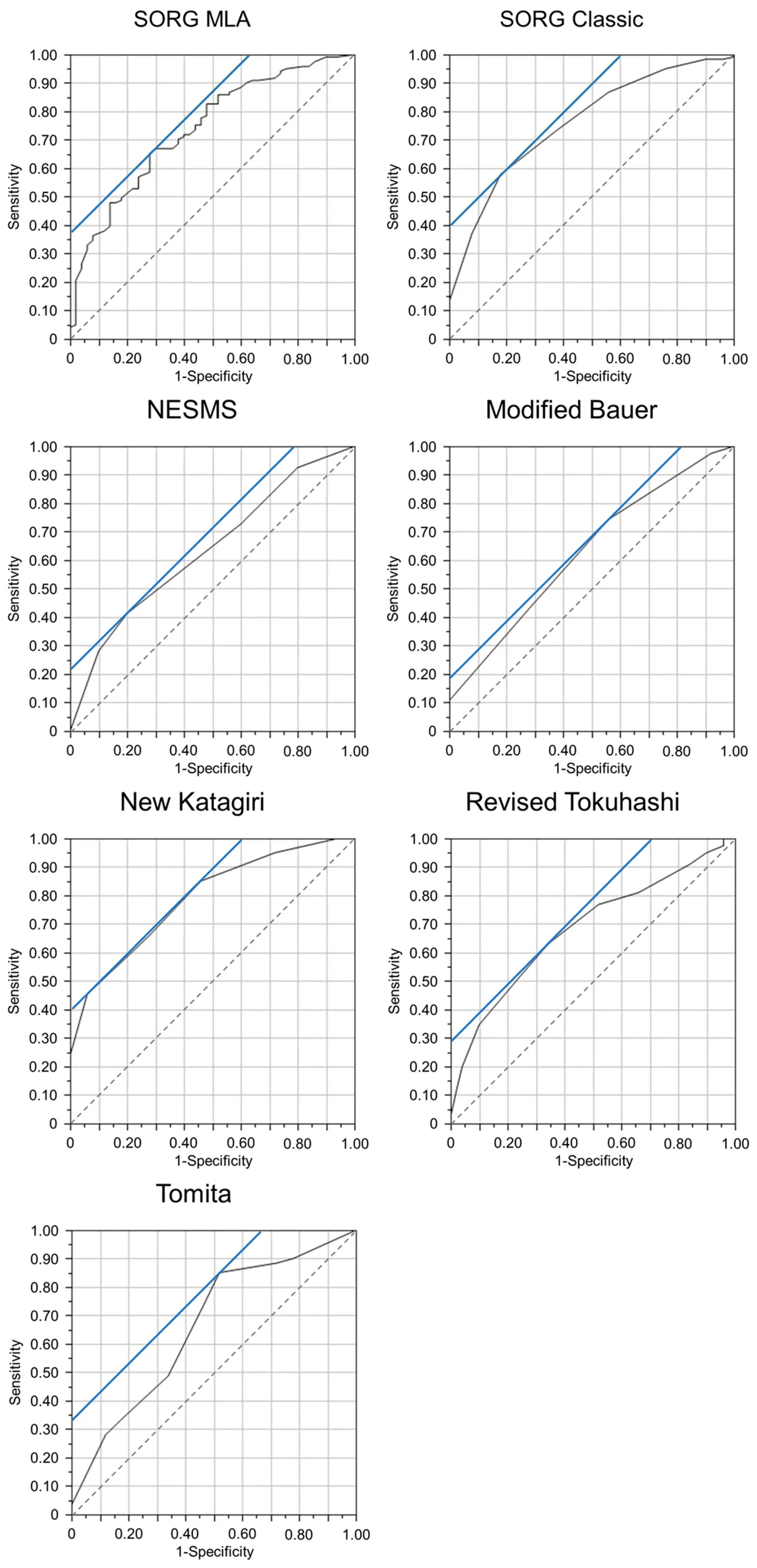
ROC curves for seven prognostic scoring systems for 1-year survival. Higher predictor values indicate a greater probability of survival. The black line represents the ROC curve, and the blue line is tangent to the ROC curve at the optimal cutoff point. The dashed diagonal line represents chance-level discrimination (AUC = 0.50).

For 1-year survival, the New Katagiri score had the highest AUC (0.79; 95% CI, 0.72–0.86), followed by the SORG classic scoring algorithm (0.76; 95% CI, 0.68–0.83) and SORG MLA (0.74; 95% CI, 0.66–0.82) (Table 5, Figure 3). After Holm adjustment, the New Katagiri score had higher AUCs than NESMS (adjusted *p* = 0.002), modified Bauer (adjusted *p* < 0.001), and Tomita (adjusted *p* = 0.01). The SORG classic scoring algorithm had higher AUCs than NESMS (adjusted *p* = 0.04) and modified Bauer (adjusted *p* = 0.02). No other pairwise comparisons were statistically significant after adjustment (Table A3). In particular, the absence of statistically significant differences among the New Katagiri score, SORG classic scoring algorithm, and SORG MLA does not demonstrate equivalence.

### 3.3. Calibration Performance of the SORG MLA

**Figure A1:**
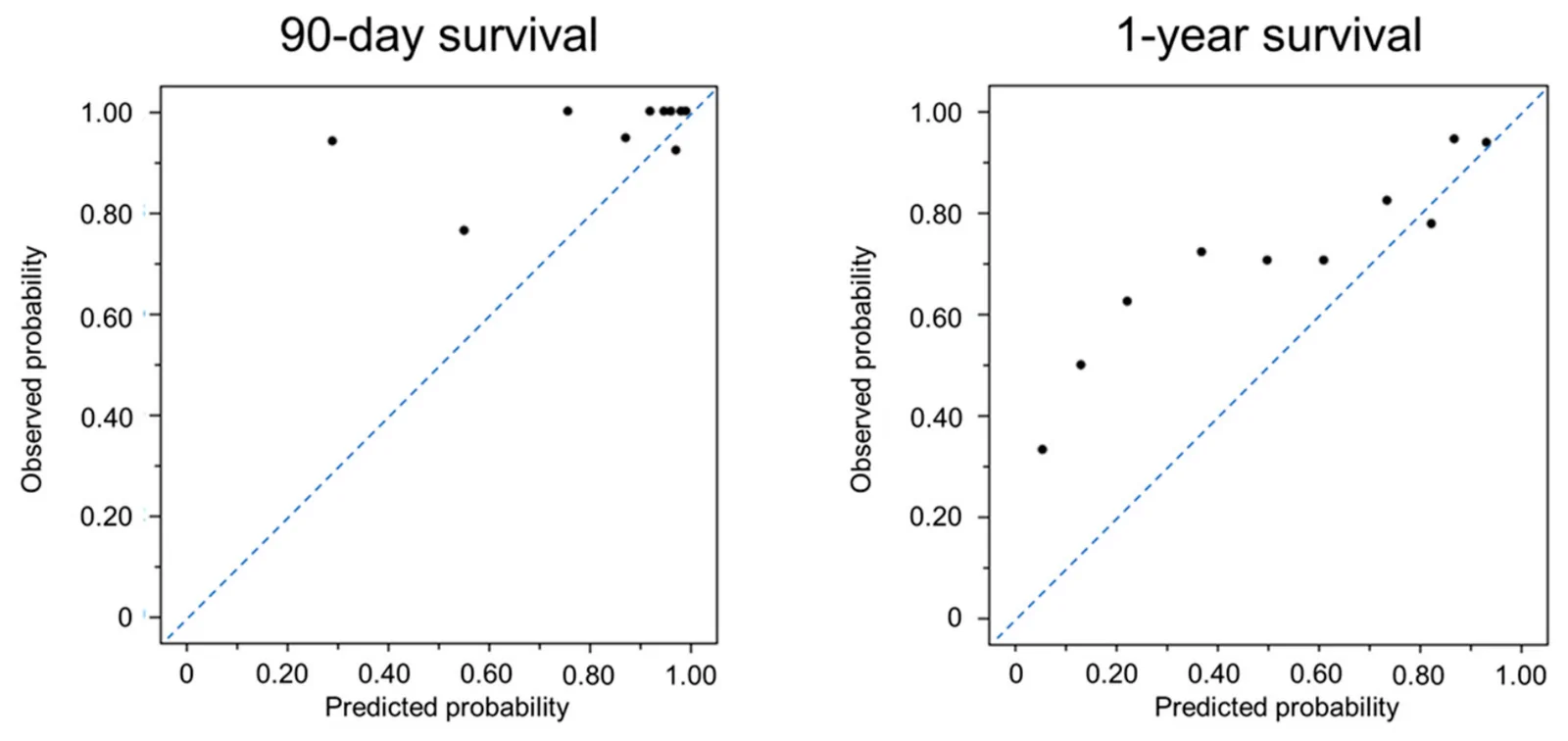
Calibration plots of the SORG MLA for 90-day and 1-year survival. Black dots indicate observed versus predicted survival probabilities across 10 groups. The dashed diagonal line represents ideal calibration.

For the SORG MLA, calibration intercepts were 2.15 (95% CI, 1.39–3.05) for 90-day survival and 1.23 (95% CI, 0.82–1.65) for 1-year survival; corresponding slopes were 0.58 (95% CI, 0.14–1.09) and 0.54 (95% CI, 0.31–0.76), respectively. Calibration plots deviated from perfect calibration at both time points (Figure A1). Brier scores were 0.088 (95% bootstrap CI, 0.063–0.115) for 90-day survival and 0.230 (95% bootstrap CI, 0.189–0.270) for 1-year survival. Thus, despite its discrimination, the SORG MLA did not yield perfectly calibrated absolute survival probabilities in this cohort. The 90-day calibration estimates should be interpreted cautiously because only seven deaths occurred, and the calibration intercept and slope may therefore be unstable.

## 4. Discussion

This study compared seven established prognostic systems in a contemporary Japanese surgical cohort. The New Katagiri score and SORG models had relatively higher AUCs than several conventional scores. After Holm adjustment, no pairwise differences were detected among the New Katagiri score, SORG classic scoring algorithm, and SORG MLA at either prediction horizon. However, this finding does not establish equivalence, particularly given the limited precision of the 90-day estimates. Accordingly, the comparison across all seven systems should be interpreted primarily as a comparison of discrimination rather than a comprehensive evaluation of clinical utility. The SORG MLA also deviated from perfect calibration. Thus, discrimination and the accuracy of absolute predicted probabilities should be considered separately. Previous external validations have similarly reported population-dependent calibration of the SORG MLA [20,21,22], warranting caution when applying absolute survival probabilities across settings.

These findings are clinically plausible in the context of advances in cancer care. Conventional systems such as the revised Tokuhashi, Tomita, and Bauer scores were developed when systemic treatment options were more limited [9,28,29]. Since then, molecularly targeted agents, immune checkpoint inhibitors, and high-precision radiotherapy have improved survival in selected patients with metastatic spinal disease [30,31,32]. These advances may limit the applicability of models developed in earlier treatment eras [13,14,15].

The New Katagiri score and SORG models incorporate variables that may better reflect contemporary prognosis, including treatment history and laboratory data. The New Katagiri score also accounts for the availability of molecularly targeted therapy for lung cancer [13,18,19]. These features may have contributed to their observed discrimination, although this study did not examine mechanisms underlying between-model differences. The relatively high AUC of the New Katagiri score may also partly reflect similarities between this Japanese cohort and the population in which the score was developed, but this hypothesis requires independent evaluation. Importantly, these findings do not demonstrate a clinical advantage of a machine-learning model over a simpler point-based score.

Median overall survival was 37 months, exceeding that reported in many earlier cohorts with metastatic spinal tumors [9,10,11]. This difference may reflect contemporary oncological treatment and selection inherent in a surgical cohort because patients undergoing surgery are not representative of all patients with spinal metastases. Prognosis and treatment response also vary substantially by primary tumor type. Tumor-specific validation was not performed because the small numbers within individual cancer categories would yield unstable estimates. Larger multicenter cohorts are needed to evaluate model performance according to primary tumor type, molecular characteristics, treatment availability, healthcare system, and treatment era.

Better discrimination does not necessarily improve surgical selection, shared decision-making, quality of life, functional independence, or other patient-centered outcomes. This study did not assess whether using any prognostic model improves these outcomes. Future validation should therefore extend beyond discrimination and calibration to decision-analytic measures such as net benefit and patient-centered endpoints. For example, the recently proposed HOME score incorporates home time in addition to overall survival [33]. Future models may also incorporate imaging biomarkers, molecular tumor characteristics, and treatment-response data, but the incremental value of greater complexity requires validation.

This study has several limitations. First, it was a retrospective, single-center study that included only surgically treated patients. These patients represent a selected subset of those with metastatic spinal tumors; therefore, the findings may not generalize to nonoperative patients or the overall population with spinal metastases. Second, 37 patients who were alive but lacked ascertainable 1-year survival status were excluded. These excluded patients had substantially poorer ECOG performance status, indicating potential spectrum and selection bias that may have affected survival estimates, AUC estimates, and the apparent comparative performance of the prognostic models. Future studies should use time-to-event validation methods that retain censored patients. Third, the sample size was determined by available cases rather than by an a priori calculation. In addition, the limited number of 90-day events resulted in wide CIs and reduced power for pairwise comparisons. Fourth, the cohort included heterogeneous primary malignancies, and tumor-specific and molecular subgroup analyses were precluded by small subgroup sizes. Fifth, systemic therapy, radiotherapy, and surgical indications evolved during the >7-year study period. An era-stratified sensitivity analysis was not performed because further subdivision would have produced sparse events and unstable AUC estimates. Nevertheless, temporal heterogeneity may have affected model performance. Sixth, the study primarily compared discrimination across seven heterogeneous systems. Calibration and Brier scores could be assessed on the original probability scale only for the SORG MLA. Decision curve analysis, net benefit, reclassification, and the effects of model-guided care on patient-centered outcomes were not assessed. Thus, these results represent comparative performance in this cohort rather than evidence of general clinical utility.

## 5. Conclusions

In this single-center cohort of surgically treated patients with metastatic spinal tumors, the New Katagiri score and SORG models showed relatively higher discrimination for postoperative survival than several conventional scoring systems. After adjustment for multiple comparisons, some differences were detected for 1-year discrimination. In contrast, no statistically significant differences were detected among the New Katagiri score, SORG classic scoring algorithm, and SORG MLA at either prediction horizon. The SORG MLA also showed imperfect calibration. Further external validation in larger multicenter cohorts is needed to assess transportability, calibration, decision-analytic utility, and patient-centered outcomes.

## Figures and Tables

**Table 1:** Patient demographics.

Factor	Value (*n* = 171)
Age (years)	64.0 ± 13.5
Male/Female	97/74
BMI (kg/m^2^)	22.3 ± 4.1
ASA-PS ≥ 3	6 (3.5%)
ECOG-PS ≥ 3	56 (32.7%)
Frankel Grade A or B	1 (0.6%)
C or D	101 (59.1%)
E	69 (40.4%)
Previous systemic treatment (+)	42 (24.6%)
Spinal metastasis ≥ 3	63 (36.8%)
Extra spinal bone metastasis ≥ 3	33 (19.3%)
Visceral/Brain metastasis (+)	61 (35.9%)

Presented as the mean with standard deviation.

**Table 2:** Preoperative laboratory values.

Factor	Value (*n* = 171)
White blood cells (×10^3^/µL)	7.0 ± 3.1
Neutrophils (×10^3^/µL)	4.9 ± 2.6
Lymphocytes (×10^3^/µL)	1.5 ± 0.7
Hemoglobin (g/dL)	13.2 ± 1.9
Platelet (×10^4^/µL)	24.9 ± 9.1
Creatinine (mg/dL)	0.79 ± 0.33
Bilirubin total (mg/dL)	0.59 ± 0.26
Alkaline phosphatase (U/L)	275 ± 220
Lactate dehydrogenase (U/L)	219 ± 105
Calcium (mg/dL)	9.2 ± 0.5
Potassium (mmol/L)	4.4 ± 0.4
C-reactive protein (mg/dL)	1.4 ± 2.9
PT-INR	0.98 ± 0.09

Presented as the mean with standard deviation.

**Table 3:** Primary tumor type.

Primary Tumor Type	Number of Patients (%)
Kidney	28 (16.4)
Lung	24 (14.0)
Thyroid gland	17 (9.9)
Sarcoma	17 (9.9)
Gastrointestinal ^1^	15 (8.8)
Breast	14 (8.2)
Prostate	12 (7.0)
Hematologic ^2^	9 (5.3)
Urothelial	5 (2.9)
Unknown	5 (2.9)
Pancreas	4 (2.3)
Hepatocellular	3 (1.8)
Bile duct	3 (1.8)
Adrenocortical	3 (1.8)
Others ^3^	12 (7.0)

^1^ Gastrointestinal: Esophageal, gastric, and colorectal cancers. ^2^ Hematologic: Lymphoma and myeloid sarcoma. ^3^ Others: Peritoneal, thymic, parathyroid, oropharyngeal, uterus, parotid gland, peripheral nerves, and skin cancers.

**Table 4:** Discrimination of the seven prognostic systems for 90-day survival. Outcome events: 7 deaths and 164 survivors.

Scoring Systems	AUC	95% CI
SORG MLA	0.77	(0.58, 0.95)
SORG Classic	0.70	(0.53, 0.87)
NESMS	0.67	(0.44, 0.89)
Modified Bauer	0.51	(0.28, 0.74)
New Katagiri	0.76	(0.60, 0.91)
Revised Tokuhashi	0.61	(0.39, 0.84)
Tomita	0.59	(0.33, 0.84)

**Table 5:** Discrimination of the seven prognostic systems for 1-year survival. Outcome events: 50 deaths and 121 survivors.

Scoring Systems	AUC	95% CI
SORG MLA	0.74	(0.66, 0.82)
SORG Classic	0.76	(0.68, 0.83)
NESMS	0.63	(0.55, 0.72)
Modified Bauer	0.63	(0.54, 0.71)
New Katagiri	0.79	(0.72, 0.86)
Revised Tokuhashi	0.68	(0.60, 0.77)
Tomita	0.66	(0.57, 0.75)

## Data Availability

Data generated and analyzed in this study are available from the corresponding author upon reasonable request.

## Supplementary Materials

The following supporting information can be downloaded at: https://www.mdpi.com/article/10.3390/jcm15207780/s1, Table S1. SORG Classic Scoring Algorithm. Total score range: 0–12 points. Higher scores indicate a poorer prognosis; Table S2. New England Spinal Metastasis Score. Total score range: 0–4 points. Higher scores indicate a better prognosis; Table S3. Modified Bauer Score. Total score range: 0–4 points. Higher scores indicate a better prognosis; Table S4. New Katagiri Score. Total score range: 0–10 points. Higher scores indicate a poorer prognosis; Table S5. Revised Tokuhashi Score. Total score range: 0–15 points. Higher scores indicate a better prognosis; Table S6. Tomita Score. Total score range: 0–8 points. Higher scores indicate a poorer prognosis; Figure S1. Flowchart of patient selection for the study cohort; Figure S2. Distributions of prognostic scores and predicted survival probabilities in the study cohort.

## Appendix A

**Table A1:** Baseline characteristics of the 37 patients excluded because 1-year survival status could not be ascertained.

Factor	Value (*n* = 37)
Age (years)	65.8 ± 14.8
Male/Female	27/10
BMI (kg/m^2^)	22.6 ± 4.6
ASA-PS ≥ 3	2 (5.4%)
ECOG-PS ≥ 3	26 (70.2%)
Frankel Grade A or B	4 (10.8%)
C or D	27 (73.0%)
E	6 (16.2%)
Previous systemic treatment (+)	5 (13.5%)
Spinal metastasis ≥ 3	17 (45.9%)
Extra spinal bone metastasis ≥ 3	10 (27.0%)
Visceral/Brain metastasis (+)	9 (24.3%)

Presented as the mean with standard deviation.

**Table A2:** Comparison of the AUCs of ROC curves for 90-day survival prediction using DeLong’s test.

Model Comparison	*p*-Value	Adjusted *p*-Value
SORG MLA vs. SORG Classic	0.44	1.00
SORG MLA vs. NESMS	0.44	1.00
SORG MLA vs. Modified Bauer	0.05	0.85
SORG MLA vs. New Katagiri	0.88	1.00
SORG MLA vs. Revised Tokuhashi	0.05	0.85
SORG MLA vs. Tomita	0.37	1.00
SORG Classic vs. NESMS	0.73	1.00
SORG Classic vs. Modified Bauer	0.006	0.11
SORG Classic vs. New Katagiri	0.30	1.00
SORG Classic vs. Revised Tokuhashi	0.39	1.00
SORG Classic vs. Tomita	0.60	1.00
NESMS vs. Modified Bauer	0.02	0.35
NESMS vs. New Katagiri	0.36	1.00
NESMS vs. Revised Tokuhashi	0.56	1.00
NESMS vs. Tomita	0.72	1.00
Modified Bauer vs. New Katagiri	0.005	0.11
Modified Bauer vs. Revised Tokuhashi	0.36	1.00
Modified Bauer vs. Tomita	0.76	1.00
New Katagiri vs. Revised Tokuhashi	0.10	1.00
New Katagiri vs. Tomita	0.40	1.00
Revised Tokuhashi vs. Tomita	0.90	1.00

**Table A3:** Comparison of the AUCs of ROC curves for 1-year survival prediction using DeLong’s test.

Model Comparison	*p*-Value	Adjusted *p*-Value
SORG MLA vs. SORG Classic	0.60	1.00
SORG MLA vs. NESMS	0.02	0.34
SORG MLA vs. Modified Bauer	0.03	0.37
SORG MLA vs. New Katagiri	0.14	1.00
SORG MLA vs. Revised Tokuhashi	0.24	1.00
SORG MLA vs. Tomita	0.12	1.00
SORG Classic vs. NESMS	0.002	0.04
SORG Classic vs. Modified Bauer	0.001	0.02
SORG Classic vs. New Katagiri	0.33	1.00
SORG Classic vs. Revised Tokuhashi	0.06	0.75
SORG Classic vs. Tomita	0.03	0.37
NESMS vs. Modified Bauer	0.84	1.00
NESMS vs. New Katagiri	<0.001	0.002
NESMS vs. Revised Tokuhashi	0.28	1.00
NESMS vs. Tomita	0.58	1.00
Modified Bauer vs. New Katagiri	<0.001	<0.001
Modified Bauer vs. Revised Tokuhashi	0.23	1.00
Modified Bauer vs. Tomita	0.33	1.00
New Katagiri vs. Revised Tokuhashi	0.007	0.12
New Katagiri vs. Tomita	<0.001	0.01
Revised Tokuhashi vs. Tomita	0.63	1.00
