# Supplementary material for: Comparative Evaluation of Prognostic Scoring Systems for Metastatic Spinal Tumors: Utility of the New Katagiri and Skeletal Oncology Research Group Models
Source: J Clin Med. 2026 Oct 9;15(20):7780. doi: 10.3390/jcm15207780 (PMC13705197; doi:10.3390/jcm15207780)
Supplement: Supplementary file 1 [file jcm-15-07780-s001.zip › jcm-4538115-supplementary.pdf]

Supplementary Table S1. SORG Classic Scoring Algorithm. Total score range: 0–12 points. Higher scores indicate a poorer prognosis.

Predictive Factor	Points
Age of ≥65 years	1
ECOG performance status (score 3 to 4)	2
Primary cancer other than lymphoma, breast cancer, multiple myeloma, kidney cancer, prostate cancer, or thyroid cancer	2
>1 spine metastasis	1
Metastases	
Lung or Liver	1
Brain (with or without lung or liver metastasis)	2
Previous systemic therapy	2
White blood-cell count of ≥11,000/μL	1
Hemoglobin level of ≤10g/dL	1

Supplementary Table S2. New England Spinal Metastasis Score. Total score range: 0–4 points. Higher scores indicate a better prognosis.

Predictive Factor	Points
Modified Bauer score ≥3	2
Ambulatory status	
Intact	1
Impaired or non-ambulatory	0
Serum albumin ≥3.5 g/dL	1

Supplementary Table S3. Modified Bauer Score. Total score range: 0–4 points. Higher scores indicate a better prognosis.

**Predictive Factor**	**Points**
No Visceral Metastases	1
No Lung Cancer	1
Solitary Skeletal Metastasis (Including Spine)	1
Primary Tumor Breast, Kidney, Lymphoma, Multiple Myeloma	1

Supplementary Table S4. New Katagiri Score. Total score range: 0–10 points. Higher scores indicate a poorer prognosis.

Predictive Factor	Points
Primary site	
Hormone-dependent breast and prostate cancer, thyroid cancer, multiple myeloma, and malignant lymphoma	0
Lung cancer treated with molecularly targeted drugs, hormone-independent breast and prostate cancer, renal cell carcinoma, endometrial and ovarian cancer, sarcoma, and others	2
Lung cancer without molecularly targeted drugs, colorectal cancer, gastric cancer, pancreatic cancer, head and neck cancer, esophageal cancer, other urological cancers, melanoma, hepatocellular carcinoma, gall bladder cancer, cervical cancer, and cancers of unknown origin	3
Visceral metastasis	
Nodular visceral or cerebral metastasis	1
Disseminated metastasis[1]	2
Laboratory data	
Abnormal[2]	1
Critical[3]	2
ECOG Performance Status 3 or 4	1
Previous chemotherapy	1
Multiple skeletal metastases	1

[1]Disseminated metastasis: Pleural, peritoneal, or leptomeningeal dissemination.

[2]Abnormal: CRP ≥ 0.4 mg/dL, LDH ≥ 250 IU/L, or serum albumin <3.7 g/dL.

[3]Critical: platelet <100,000/lL, serum calcium ≥10.3 mg/dL, or total bilirubin ≥1.4.

Supplementary Table S5. Revised Tokuhashi Score. Total score range: 0–15 points. Higher scores indicate a better prognosis.

Predictive Factor	Points
General Condition (KPS)	
Poor (KPS 10%-40%)	0
Moderate (KPS 50%-70%)	1
Good (KPS 80%-100%)	2
Number of Extraspinal Bone Metastases	
≥3	0
1-2	1
0	2
Number of Metastases in the Vertebral Body	
≥3	0
2	1
1	2
Metastases to the Major Internal Organs	
Unremovable	0
Removable	1
No Metastases	2
Primary Site of Cancer	
Lung, Osteosarcoma, Stomach, Bladder, Esophagus, Pancreas	0
Liver, Gallbladder, Unidentified	1
Others	2
Kidney, Uterus	3
Rectum	4
Thyroid, Prostate, Breast, Carcinoid Tumor	5
Spinal Cord Palsy	
Complete (A,B)	0
Incomplete (Frankel C,D)	1
None (Frankel E)	2

Supplementary Table S6. Tomita Score. Total score range: 0–8 points. Higher scores indicate a poorer prognosis.

Predictive Factor	Points
Primary Tumor	
Slow growth (breast, prostate, thyroid, etc.)	0
Moderate growth (kidney, uterus, etc.)	1
Rapid growth (lung, liver, stomach, colon, primary unknown, etc.).	2
Visceral Metastases	
Treatable	2
Untreatable	4
Bone Metastases	
Solitary or Isolated	1
Multiple	2

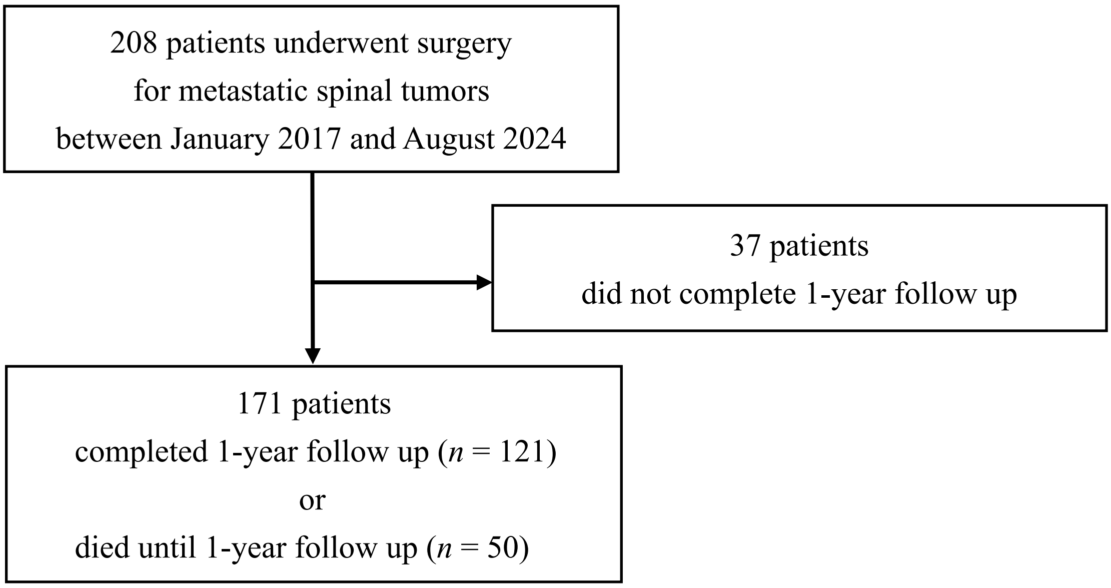

Supplementary Figure S1 Flowchart of patient selection for the study cohort.

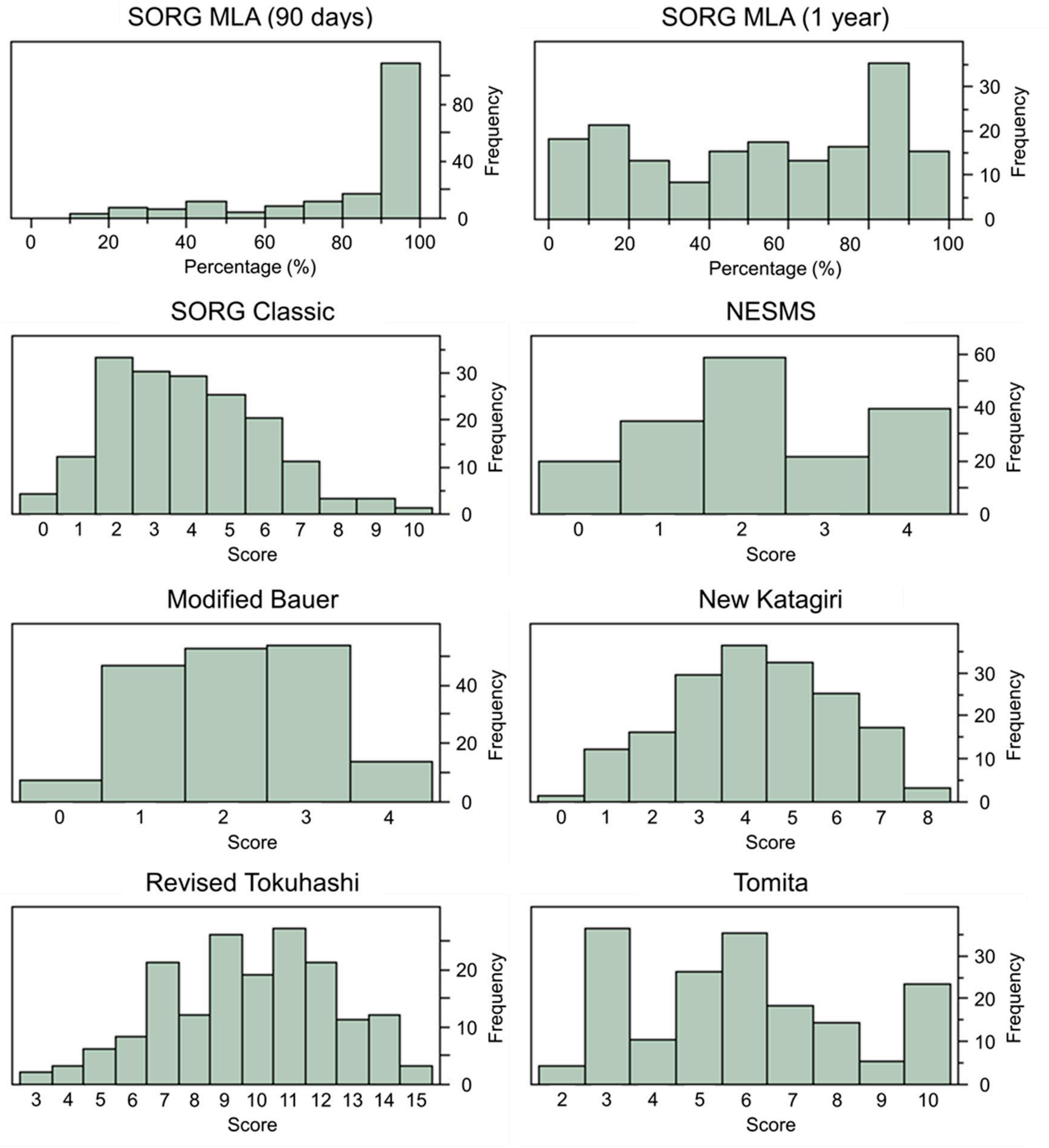

Supplementary Figure S2. Distributions of prognostic scores and predicted survival probabilities in the study cohort.
